# Neural Representation of the English Vowel Feature [High]: Evidence From /ε/ vs. /ɪ/

**DOI:** 10.3389/fnhum.2021.629517

**Published:** 2021-04-09

**Authors:** Yan H. Yu, Valerie L. Shafer

**Affiliations:** ^1^Department of Communication Sciences and Disorders, St. John’s University, Queens, NY, United States; ^2^The Graduate Center, City University of New York, New York, NY, United States

**Keywords:** mismatch negativity, late discriminative negativity, brain asymmetry, underspecification, vowel, event-related potential, prior, predictive modeling

## Abstract

Many studies have observed modulation of the amplitude of the neural index mismatch negativity (MMN) related to which member of a phoneme contrast [phoneme A, phoneme B] serves as the frequent (standard) and which serves as the infrequent (deviant) stimulus (i.e., AAAB vs. BBBA) in an oddball paradigm. Explanations for this amplitude modulation range from acoustic to linguistic factors. We tested whether exchanging the role of the mid vowel /ε/ vs. high vowel /ɪ/ of English modulated MMN amplitude and whether the pattern of modulation was compatible with an underspecification account, in which the underspecified height values are [−high] and [−low]. MMN was larger for /ε/ as the deviant, but only when compared across conditions to itself as the standard. For the within-condition comparison, MMN was larger to /ɪ/ deviant minus /ε/ standard than to the reverse. A condition order effect was also observed. MMN amplitude was smaller to the deviant stimulus if it had previously served as the standard. In addition, the amplitudes of late discriminative negativity (LDN) showed similar asymmetry. LDN was larger for deviant /ε/ than deviant /ɪ/ when compared to themselves as the standard. These findings were compatible with an underspecification account, but also with other accounts, such as the Natural Referent Vowel model and a prototype model; we also suggest that non-linguistic factors need to be carefully considered as additional sources of speech processing asymmetries.

## Introduction

The ability to discriminate and categorize speech sound contrasts is crucial for fast and efficient lexical access, but our understanding of how this process unfolds is still incomplete. An enduring question has been how speech sound information is represented in the human mind/brain to support the process of lexical access (Strange, 2011). Two general proposals have been offered. One proposal is that phonological representations precisely encode the physical world (in terms of sensory, motor, and statistical properties, such as lexical type and token frequency) (Bybee, 2002). The alternative is that phonological representations are abstract in nature and that the information that is part of the representation is determined by constraints that cannot be fully explained using sensory and motor factors or other information external to the phonological system. Models arguing for abstract representations have favored reducing speech sounds to a small set of features (often binary) that are assumed/hypothesized to reflect what is stored as part of the representation (Chomsky and Halle, 1968). These “abstract” models also favor representations that have the minimum necessary information, with predictable or redundant information being filled in by some process during production and perception. No consensus has yet been reached regarding which model is superior, perhaps because both types of models have found some support. Much of the evidence bearing on these questions has come from cross-linguistic studies that examined the patterning of phonological systems, or from behavioral psycholinguistic studies (Kazanina et al., 2018; Samuel, 2020).

Until fairly recently, few studies have used neurophysiological methods to directly address the nature of phonological representations. Initially, these methods were designed to test questions of phonetic representations, such as categorical perception or questions asking whether particular brain measures, such as mismatch negativity (MMN) or P3b indexed acoustic (auditory general), phonetic (language general), or phonemic (language specific) processes (Buchwald et al., 1994; Näätänen et al., 1997; for review, Näätänen et al., 2007).

Mismatch negativity has emerged as the primary method for testing questions of neural representation of speech. MMN is a neural discriminative response generated in the auditory cortex in response to a discriminable change in a repetitive auditory stimulus, which can be generated with or without attention (Alho, 1995; Näätänen et al., 2007, 2019). The current understanding of the processes underlying MMN is that the repeated stimulus or stimulus pattern (standard) leads to construction of a central sound representation. This standard representation is then used to predict subsequent stimuli, and on encountering a stimulus that diverges from the prediction (deviant), the MMN is generated (Näätänen et al., 2011). Both within block MMN (deviant stimulus minus standard stimulus from the same block) and identity MMN (iMMN) (the stimulus used as the deviant in one condition minus the same stimulus used as the standard in the other condition) have been widely used in the literature. The iMMN response isolates the contextual effects of sound discrimination because it eliminates the difference in the auditory evoked potential (AEP) that is attributable to low-level acoustic differences in spectral, temporal, and intensity information (Jacobsen and Schröger, 2001, 2003; Kujala et al., 2007; Möttönen et al., 2013).

The Eulitz and Lahiri (2004) paper was, perhaps, the first study using neurophysiology that was directly designed to test a phonological model of representation. They tested the Featurally Underspecified Lexicon (FUL) model and used evidence from an asymmetric discrimination pattern of the MMN to test predictions derived from the model. In FUL, all phonetic features in the surface form are extracted from the acoustic signal, but at the level of the mental lexicon, some speech sounds will be underspecified for certain features (Eulitz et al., 1995; Lahiri and Reetz, 2002; Eulitz and Lahiri, 2004). Unspecified features are then filled in by other means because they are predictable. Eulitz and Lahiri (2004) predicted that presenting the vowel with the underspecified feature (in this case, default [+coronal]) as the standard stimulus, and the stimulus with the specified feature (in this case explicitly specified as [−coronal]) as the deviant stimulus would result in a smaller MMN. In other words, no conflict would be observed because no feature value is specified in the neural representation constructed to the standard for the underspecified member of the pair. They observed a smaller MMN to the vowel contrast when the underspecified member of the pair served as the standard. Since this paper, several other studies have tested the viability of underspecification models and generally have found support (Cornell et al., 2011, 2013; Scharinger et al., 2012; Hestvik and Durvasula, 2016; Cummings et al., 2017; Scharinger and Samuels, 2017; Højlund et al., 2019).

A number of studies that pre-dated Eulitz and Lahiri (2004), and that used MMN have observed patterns that bear upon the question of phonological asymmetry, even though they were not designed to specifically test a specific phonological theory. The earliest studies focused on questions related to categorical perception (e.g., Aaltonen et al., 1987, 1997; Sams et al., 1990; Maiste et al., 1995; Näätänen et al., 1997; Sharma and Dorman, 1998) or to auditory physiology (e.g., Kraus et al., 1992). The design of these first speech experiments was informed by a fairly large number of studies using MMN to examine auditory processing of non-speech features such as frequency, duration, and pitch (e.g., Kaukoranta et al., 1989; Nyman et al., 1990; Ritter et al., 1992; see Näätänen et al., 2007 for review).

Several of these early studies noticed and commented on the asymmetrical results of the MMN related to which auditory sound served as the standard and which served as a deviant. The practice of “flipping” the role of the two stimuli of interest was initially undertaken so that the event-related potential (ERP) to the deviant stimulus could be compared to the ERP to the same stimulus when it served as the standard (e.g., Sharma et al., 2004). The initial motivation for this exchange (e.g., flip-flop) was to control for differences in AEPs that indexed difference in timing of acoustic features. For example, the N1 amplitudes were larger for the vowel onset following a long-lag voice onset time (VOT) consonant than that following a short-lag VOT (Toscano et al., 2010), and VOT has also been shown to influence the latency of the N1 (M100) (Frye et al., 2007). In addition, different spectral properties of a stimulus will engage different neural populations in the primary auditory cortex. The longer inter-deviant interval compared to the inter-standard interval would then allow for greater recovery from neural refractoriness, which can be seen as an increased negativity of the N1b (e.g., McGee et al., 1997; Sharma et al., 2004). The question of what should serve as the control condition in the MMN paradigm to minimize these acoustic effects has sporadically been addressed, but is not directly a concern of the current paper (e.g., Cowan et al., 1993; Phillips et al., 1995; McGee et al., 1997; Sharma et al., 2004; Scharinger et al., 2012, 2016; Hestvik and Durvasula, 2016).

What is relevant for the current paper is that these first studies often observed an asymmetry in the amplitude of the MMN dependent on which of two contrasts served as the standard and which served as the deviant (e.g., Maiste et al., 1995; Shafer et al., 2004). For example, using a continuum of nine stimuli, Maiste et al. (1995) observed a clear MMN to cross-category stop consonant place deviants [da] only when [ba] served as the standard. Flipping the standard and deviant so that [da] was the standard stimulus resulted in no MMN, even to the phonetically most different stimulus [ba]. Maiste et al. (1995) attributed this finding to acoustic factors rather than to phonetic/phonological properties. Specifically, [da] showed a broader spectrum than [ba], and [da] contained the frequencies of the [ba] onset. But their finding of asymmetry is also compatible with the FUL model claim, in which [coronal] is the underspecified feature.

Shafer et al. (2004) observed a similar asymmetry to the Maiste et al. (1995) finding, but they suggested that the asymmetry was related to the linguistic property called markedness. They observed a larger MMN to the bilabial [ba] as a standard than to the dental or retroflex stop [da] as a standard (from a continuum of 10 stimuli from bilabial [ba] to retroflex [da]). Cross-linguistic surveys have observed that the retroflex category is less common and have characterized this as more “marked” (Maddieson, 1984), but this was a *post hoc* explanation. As pointed out by Haspelmath (2006), invoking markedness is not a satisfactory explanation, in part because the term has many different meanings in linguistics, but also because it is only a relabeling of the observation that the retroflexed category is somehow difficult to discriminate/produce compared to the bilabial category. In addition, the Shafer et al. (2004) study found that language experience modulated the asymmetry effect in that Hindi compared to naïve American English listeners showed a larger and earlier MMN to the [ba] deviant in the context of the retroflex standard.

Predictions derived from categorical perception (the original motivation for the study) cannot account for the cross-linguistic difference observed in Shafer et al. (2004), since for both groups the bilabial and retroflexed stops are perceived as distinct categories, as shown in the identification behavior of the participants in the study. The findings are compatible with either the acoustic explanation of Maiste et al. (1995) or with the underspecification approach of Eulitz and Lahiri (2004). Specifically, [coronal] (referring to the tongue tip/blade articulator) is argued to be the underspecified feature in many languages (Kiparsky, 1985; Paradis and Prunet, 1991), and if we accept this analysis for both Hindi and English, then the bilabial [ba] must be explicitly marked (that is [−coronal]). It is important to note that arguments used to determine which feature is underspecified in a language or across languages often make use of the notion of markedness.

Despite these findings consistent with linguistic accounts for asymmetry, it is important to fully consider psychophysical explanations. A few studies have demonstrated larger MMN to frequency increments compared to decrements using non-speech tones (e.g., Peter et al., 2010; Shiramatsu and Takahashi, 2018). The asymmetrical pattern observed for /ba/ and /da/ are consistent with this finding because /da/ has a higher frequency F2 formant onset than /ba/.

Asymmetries associated with speech sound length features have also been observed (e.g., Kirmse et al., 2007; Hisagi et al., 2010, 2015b; Chládková et al., 2015), and in some studies, have been attributed to psychophysical properties. However, the findings for speech are somewhat mixed. Several studies have observed a larger MMN to length increases for vowels or consonants irrespective of the phonological status of [length] in the language (Kirmse et al., 2007; Hisagi et al., 2010). The explanation for this finding was that duration increment is easier to discriminate than decrement, and that this pattern is acoustic in nature because it has also been observed for non-speech auditory information (Takegata et al., 2008). Findings from investigations of languages where length is a secondary cue for phonemic contrast (e.g., English and Dutch), however, suggest that phonological factors better explain asymmetries (Chládková et al., 2015; Shafer et al., accepted). For example, the study by Chládková et al. (2015) found evidence of an asymmetry only for the longer vowel /a:/, but not for the shorter /ɑ/ and suggested that this was consistent with specification of the long duration (and the short vowel being unspecified).

Another explanation that has been offered for asymmetries in speech sound processing and which could be extended to the findings in the MMN literature is the Natural Referent Vowel (NRV) framework proposed by Polka and Bohn (2003, 2011). According to the NRV, discrimination is easier when the reference (standard) is more centralized in the articulatory space compared to the change (deviant). This model makes similar predictions to Kuhl’s perceptual magnet model, in which the better exemplar (prototype), when serving as the reference leads to poorer discrimination of a less prototypical vowel of the same category (in this case /i/) (Kuhl et al., 1992). Thus, both the NRV and the Perceptual Magnet model predict that MMN would be smaller when a more peripheral (prototypical) vowel serves as the reference (standard) compared to a more central (less prototypical) vowel. Two studies using MMN show support for the Perceptual Magnet effect (Aaltonen et al., 1997; Sharma and Dorman, 1998), although the pattern was modulated by whether a listener was good or poor at categorizing the speech stimuli in the study by Aaltonen et al. (1997).

A different explanation for asymmetries in MMN amplitude is related to experimental design. In a series of experiments, MMN was shown to be attenuated to a deviant stimulus if it had previously served as a standard (e.g., Todd et al., 2014). In addition, brief blocks that alternate which stimulus is the standard in the first half and which is the deviant in the second half can result in even greater attenuation of the MMN (Todd et al., 2014). Thus, it is possible that asymmetries could be an artifact of the order effect for any study that did not carefully counterbalance condition order.

### The Present Study

The current study was designed to further address whether neurophysiological evidence is compatible with a model of underspecification. As we noted above, many studies that observed asymmetries in MMN did not predict these patterns, and rather, offered *post hoc* explanations. Thus, in the current study, we first looked for independent evidence that would allow predictions to be made with regards to the vowel contrast /ε/ vs. /ɪ/. We chose this contrast because we have used it to test a range of questions related to infant development, child language disorders and second language learning (e.g., clinical population: Shafer et al., 2005; Datta et al., 2010; developmental populations: Shafer et al., 2010, 2011; Yu et al., 2019; second language: Hisagi et al., 2015a; Datta et al., 2020). In all of these papers, which focused on group differences, /ε/ served as the standard and /ɪ/ as the deviant (and this order was selected based on pilot data that indicated a larger MMN for this direction; see Morr, 2002). The stimuli consisted of a resynthesized naturally produced vowel in which the F1 and F2 formants were edited to create a nine-step continuum from /ε/ to /ɪ/. Previous studies with adults and children indicated that step 3 and step 9 were consistently identified as /ε/ and /ɪ/, respectively and that they were both the same distance from the perceptual boundary (at step 6 on the continuum) (Shafer et al., 2005; Datta et al., 2010; Hisagi et al., 2015a).

We chose to use this simple paradigm of one token per category rather than using multiple tokens (e.g., Eulitz and Lahiri, 2004; Hisagi et al., 2010; Hestvik and Durvasula, 2016; Yu et al., 2017, 2018) because we wanted to be able to directly relate the findings to the previous studies where we used these stimuli. Importantly, speech sounds, whether presented as a single token, or as one of a set of tokens are processed at a phonological level. Research suggests that listeners automatically extract native-language phonetic features to allow for phoneme categorization (if the auditory information is sufficiently speech-like) (Strange, 2011). In the case of the discrimination process indexed by MMN, listeners rely on automatic selective perception routines, which reflect the native language phonological categories (Hisagi et al., 2010; Yu et al., 2017; Shafer et al., accepted). MMN will also index discrimination on the basis of acoustic factors, which can complicate interpretation. However, our study comparing MMN to this vowel contrast between monolingual English speakers and bilingual Spanish–English speakers clearly indicated that these vowel stimuli engage phonological processing (Hisagi et al., 2015a).

The current study was designed to examine whether there is an asymmetry to this vowel contrast in native English-speaking adults. Several studies lead to the claim that /ε/ is the underspecified vowel of the pair. Stemberger (1991a, b) found evidence from speech errors supporting that English /ε/ is the underspecified vowel (with underspecification of [−high] [−low] [−back] [−round]). Stemberger (1992) compared the mispronunciation rates of vowel contrasts involving [high] and [back] features (e.g., /ɪ/-/ε/, /ɪ/-/ʌ/, /ε/-/ʌ/). He observed significantly more errors involving the non-high vowel /ε/ being replaced by the high vowel /ɪ/ than the other way around. He also examined whether type frequency (number of English words with a specific phoneme) would account for the error pattern. If so, vowels with higher type frequency should be more resistant to speech errors. His findings, however, were incompatible with this word frequency account. Another study using MMN also supports the claim that /ε/ is underspecified (Scharinger et al., 2012). They tested the proposal that [high] and [low] features of vowels are specified in the underlying representation, and thus /ε/, as a mid vowel, has no specification for these values. They observed a larger MMN when a low vowel /æ/ served as the standard and /ε/ as the deviant compared to the MMN to the contrast presented in the reverse direction. In addition, they observed no asymmetry in the MMN for /ɪ/ vs. /æ/, which both are marked for height in the model, but for different features ([high] for /ɪ/ and [low] for /æ/).

Taken together, these results lead to the hypothesis that the MMN will be larger when /ɪ/ serves as the standard and /ε/ as the deviant because /ɪ/ is marked as [+high] and /ε/ has no height specification. The NRV model predicts a larger MMN when the more peripheral vowel /ɪ/ serves as the standard. The acoustic account does not lead to a clear prediction that one order will show a larger MMN because vowels are both the same in duration; the spectrum of these two vowels differs minimally. In considering the direction of frequency change (in terms of increment or decrement), F2 is higher for /ɪ/ than /ε/, but the reverse pattern is found for F1. Thus, the two directions of change conflict with regards to predicting direction of asymmetry. We also examined whether the order of presentation of the conditions would modulate the MMN, predicting a smaller MMN to the vowel which was first presented as a standard. Finally, we performed analysis comparing the deviant stimulus to the standard within a condition (cross-block MMN, comparing ERPs to two acoustically-different stimuli) and comparing the deviant to the standard across conditions (iMMN from the identity stimuli, where the ERPs are to the same stimulus serving as the deviant in one condition and the standard in the other condition). We chose to undertake the analysis in both ways to allow an explicit comparison of how these different methods affect the MMN and to allow us to relate our findings here to our previous studies.

## Materials and Methods

### Participants

Twenty young adult participants between the ages of 19 and 27 years old were recruited and provided written informed consent. Data from two participants were collected using the incorrect sampling rate, and data from one participant were too noisy. Data from the remaining 17 participants (Male = 5) were included in the statistical analyses. All participants passed a standard hearing screening in the laboratory and reported no history of hearing/speech-language/neurological/developmental impairment. All participants were native English speakers, seven of them were monolingual English speakers, and ten had some Spanish exposure from family and/or had taken regular school Spanish as second language classes, but all reported dominance in English. Each participant was paid $10 per hour for participation. The study was approved by the human subject research institutional review board at St. John’s University, New York, and was conducted in compliance with the Declaration of Helsinki.

### Stimuli

**FIGURE 1:**
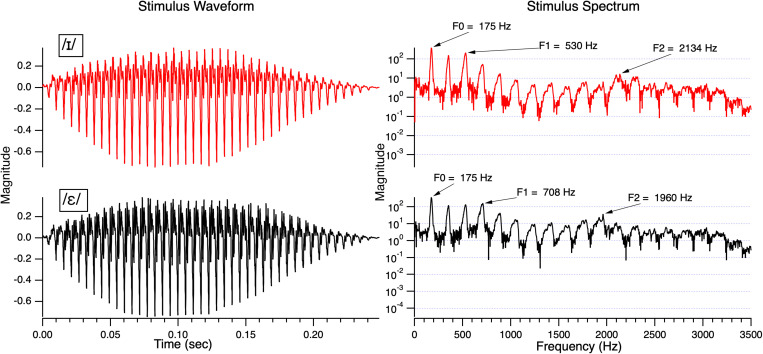
The waveforms of /ɪ/ and /ε/ on the left and the corresponding spectra on the right. The stimuli were resynthesized and edited to retain the natural bandwidth of each formant and the natural change in F0 across the stimulus (higher at the onset and falling off at the offset).

Two English vowels /ɪ/ (as in the word *bit*) and /ε/ (as in the word *bet*) were used in the study. To create the vowels, a natural token of a neutral vowel /ʌ/ was produced by a female with an F0 of approximately 190 Hz. This vowel was resynthesized and edited using target formant frequencies based on natural productions of /ɪ/ and /ε/ from the same speaker using Analysis by Synthesis Lab, version 3.2 (see Shafer et al., 2010, 2011 for details). A nine-equal-step continuum was created using equal steps for the first formant (F1) and second formant (F2). The two tokens for this study were Step 3 and Step 9 on the continuum and were selected to be equidistant from the boundary (determined in piloting). These stimuli had the following mean center frequencies: for Step 3, F1 = 500, F2 = 2160; for Step 9 F1 = 650 Hz, F2 = 1980 Hz. The two stimuli had an identical duration of 250 ms and identical third (F3 = 2174) and fourth (F4 = 3175) formants. Step 3 was identified as /ɪ/ and Step 9 as /ε/, respectively, by both monolingual English-speaking children and adults in studies from our laboratory (e.g., Datta et al., 2010; Hisagi et al., 2015a). Figure 1 shows the waveforms and power spectrum of the two stimuli.

### The ERP Paradigm

The stimuli were presented using over-the-ear headphones at a comfortable listening volume. Two blocks (one condition per block) of 1000 stimuli (20% deviant) were presented in a counter-balanced order across participants at the rate of 650 ms (interstimulus interval of 400 ms). In one condition Step 3 /ɪ/ was the standard, and Step 9 /ɪ/ was the deviant, and in the other condition the standard and deviant were switched. Thus, a total of 200 deviant and 800 standard trials were delivered for each stimulus. The data were recorded in an electrically shielded and sound-attenuated booth, and the participants were watching a muted movie with captions played over a handheld tablet during the data recording for the purpose of keeping the participants occupied and directing the participants’ attention away from the auditory speech sounds. All the participants watched the same movie. This procedure is commonly used in MMN designs because it engages the participant’s attention for the long period of time needed to obtain a sufficient number of trials for good signal to noise ratio.

The experiment was programmed with the E-prime 2 Professional software (version 2.0.10.356) (Psychology Software Tools) to deliver stimuli. The data were acquired and digitized via Netstation software version 5.4. The 65-channel HydroCel sensor nets from Electrical Geodesics, Inc., 400 system, using Ag/AgCl plated electrodes housed in electrolyte-soaked sponges were placed on the participant’s scalp. The impedances of the electrodes were kept at or below 50 kΩ. The EEG was recorded using a bandpass filter of 0.1–100 Hz and sampling rate of 1000 Hz with Cz as the reference electrode. The continuous EEG waveforms were processed offline, using a bandpass filter of 0.3–30 Hz in Netstation version 5.4, and were then segmented into epochs of 200 ms pre-stimulus and 800 ms post-stimulus periods. BESA Research 6.0 (BESA GmbH, 2014) was used for further offline processing. Automatic artifact correction was applied to each participant’s data using an HEOG threshold of 150 μV and a VEOG threshold of 250 μV for eye movement noise, and thresholds for bad channels were set at 120 μV for amplitude (gradient of 75). The individual averaged data were referenced to an average reference. There was no significant difference in the number of accepted trials by condition (/ɪ/ deviant: mean = 173 trials, SD = 18; /ε/ deviant: mean = 175 trials, SD = 21; /ɪ/ standard: mean = 505 trials, SD = 64; /ε/ standard: mean = 510 trials, SD = 46; *p*-values > 0.74).

### ERP Analysis

The data were downsampled to 20 ms per data point (after filtering using the engineer’s Nyquist). Permutation analyses were used to control the multiple comparison problems that commonly arise in parametric statistical procedures (e.g., multiple *t*-tests, analysis of variance) when these involve a large number of statistical comparisons (e.g., multiple correlated sensor sites and correlated time points) (Maris and Oostenveld, 2007). This approach reduces the rate of false positives (Type I error) in ERP data analyses (Lage-Castellanos et al., 2010). Permutation tests also have the advantage of making no assumptions about the distribution of the data. The test was performed in Rstudio using the RVAideMemoire package.

To examine the features of MMN, we utilized a two-step sequential temporo-spatial principal component analysis (PCA) to determine the time window of analysis and electrodes to include for analysis (Dien, 2010). We then used the time window and electrode sites obtained from the PCA to examine the MMN amplitude effects. The two-step sequential temporo-spatial PCA has the advantage of objectively identifying time windows and electrode regions for examining the target effects (Dien and Frishkoff, 2005; Dien, 2010, 2012). This is a mathematical way to isolate the underlying latent ERP components in the temporal and spatial domain so that there is no need to subjectively select the time window and electrode sites for further analysis (Luck and Gaspelin, 2017). We used the difference waves as the input for the PCA to better focus on the temporal and spatial features of the mismatch itself (Handy, 2005; Hestvik and Durvasula, 2016). Based on the PCA results, we averaged the amplitudes of the electrode sites for each temporal-spatial component, and adopted six time-bins of 20 ms, centered around the peak of each temporal-spatial component as the time of interest. Permutation ANOVAs using stimulus condition (/ε/ vs. /ɪ/) and time as the independent variables were performed to determine the main effects of stimulus condition and the interaction between the two variables. The iMMN was generated by subtracting the standard from the deviant of the same stimulus (deviant /ɪ/ –standard /ɪ/; deviant /ε/ –standard /ε/). Our main focus was the iMMN. We also examined the identity late discriminative negativity (LDN) based on the results of the PCA. Finally, we examined the MMN asymmetry within the same block and presentation order effect to allow our findings to be directly related to our prior studies and the NRV model; to do this, MMN was generated by subtracting the standard from the deviant in the same block (deviant /ε/ –standard /ɪ/; deviant /ɪ/ –standard /ε/).

The main effect of stimulus was followed up by a permutation Student’s *t*-test. Permutation ANOVAs and permutation Student’s *t*-tests were also used to examine whether there was any order effect between those who heard /ε/ as the standard first vs. those who heard /ɪ/ as the standard first using the iMMN. We chose to include this factor because a number of studies have shown attenuation of the MMN to the deviant if it previously had occurred as a standard (McGee et al., 2001; Todd et al., 2014). Nine participants heard /ε/ as the standard first, and eight participants heard /ɪ/ as the standard first.

## Results

### Results of the PCA for Identity MMN/LDN (Deviant /ε/–Standard /ɪ/; Deviant /ɪ/–Standard /ε/)

The goal of the PCA was to identify a set of sites and the time range that contributed to the MMN and LDN that would not be biased for one analysis approach. These sites would then be used to construct unbiased measures to test the question of whether MMN and LDN amplitudes are different depending on which stimulus serves as the standard and which serves as the deviant.

The analyses were performed using the EP tools by Dien (2010). First, a temporal Promax rotation with a covariance relationship matrix and Kaiser weighting (kappa = 3) was performed followed by the Scree test in combination with the Parallel Test (Horn, 1965), which compares the Scree of the dataset to that generated from a fully random dataset. We retained components that had a total variance larger than 5%; only the first four temporal components met this criterion. For the temporal PCA, 25 temporal components were retained, which accounted for a total variance of 81%, with the first four components each having variance accounted for greater than 5%. The time windows and variances accounted for (in parenthesis) for these four components are TF1 at 520 ms (23%), TF2 at 781 ms (15.6%), TF3 at 297 ms (10.4%), and TF4 at 159 ms (6.5%). These temporal factors were submitted to the spatial infomax rotation, which resulted in five spatial factors for each temporal component.

The TF2 factors were discarded because they were outside the time window of MMN. Only TF1SF5, TF3SF3, and TF4SF3 showed a spatial distribution with maxima at the fronto-central regions. TF1SF5 showed the maximal negativity at site 51 (slightly anterior to the midpoint of Cz and C4) with inversion near site 55 (adjacent to the mastoid). The sites that had factor loadings >0.6 for TF1SF5 were only sites 51 and 41. TF3SF3 showed the maximal negativity at site 4 (FCz) and inversion at site 10 (Fp1). The sites that had factor loadings >0.6 for TF3SF3 were 4, 7, 16, 20, 41, 50, 51, 53, 54, and 65. TF4SF3 showed the maximal negativity at Cz, which is slightly posterior to what is expected for MMN, but it was the only factor that was in the temporal window of MMN and that also showed contribution from frontal sites and inversion at inferior sites (maximal inversion at site 1/F10). The sites that had factor loadings >0.6 for TF4SF3 were 4, 7, 15, 16, 41, 50, 51, 53, 54, and 65, which were highly similar to the sites for TF3SF3. Therefore, three PCA components (TF4SF3, TF3SF3, and TF1SF5) were selected, and sites with loadings >0.6 for each component were averaged to derive one measure corresponding to each of the three PCA components and then used in the subsequent statistical analyses. TF4SF3 peaked at 159 ms, which fell within the typical timeframe for MMN and TF1SF5 peaked at 520 which fell within the LDN time window reported in prior literature. The TF3SF3 peaked at 297 ms, falling in a later time frame than generally reported for MMN, and somewhat early for LDN. We selected the time window for each derived measure to correspond to the onset and offset latencies where the amplitude was 1/2 of the peak amplitude value observed for the PCA component. Because we downsampled by intervals of 20 ms, the onset and offset of the selected interval was the nearest value (for example, if the 1/2 amplitude onset was 109 ms, then the interval onset was 100 ms).

### Identity MMN and LDN Results

#### TF4SF3-Derived Measure

**FIGURE 2:**
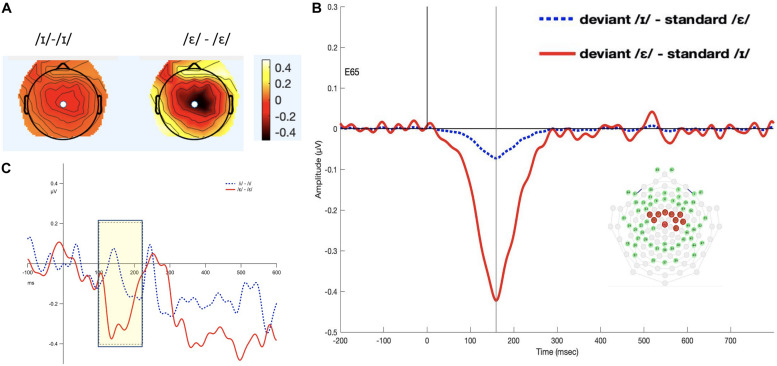
Panel **(A)** shows the topomap of the principal component TF4SF3. Panel **(B)** shows the waveforms of the iMMNs from the principal component TF4SF3. The blue dotted line indicates the /ɪ/ condition, and the red solid line indicates the /ε/ condition. The PCA component peaked at 159 ms, and the sites with loading larger than 0.6 were 4, 7, 15, 16, 41, 50, 51, 53, 54, and 65 (electrodes marked red on the figure of the EGI 65 channel sensor net). Panel **(C)** shows the waveforms of the scalp event-related potentials for the iMMNs for TF4SF3. The blue dotted line indicates the /ɪ/ condition, and the red solid line indicates the /ε/ condition. Time window of statistical analysis is marked with a shaded rectangle.

The 100–220 ms interval was selected (see Figure 2). The permutation ANOVA using condition (/ε/ vs. /ɪ/) and time (six, 20-ms time bins) as the independent variable revealed a significant main effect of condition (*F*_1_,_192_ = 3.962, *p* < 0.05). The time main effect (*F*_5_,_192_ = 0.3676, *p* = 0.88) and time by condition interaction (*F*_5_,_192_ = 0.9577, *p* = 0.44) were not significant. Follow-up permutation Student’s *t*-test on the condition effect revealed that the amplitude of MMN for /ε/ was significantly more negative than for /ɪ/ (*t* = 5.424, *p* < 0.001).

#### TF3SF3-Derived Measure

**FIGURE 3:**
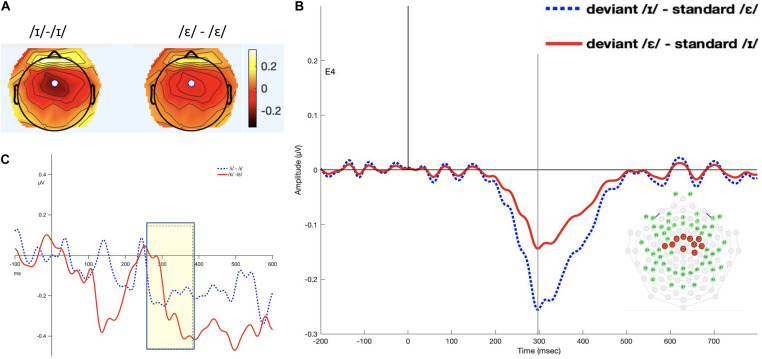
Panel **(A)** shows the topomap of the principal component TF3SF3. Maximal negativity is at site 4 (white dot). Panel **(B)** shows the waveforms of the iMMNs from the principal component TF3SF3. The blue dotted line indicates the /ɪ/ condition, and the red solid line indicates the /ε/ condition. The PCA component peaked at 297 ms, and the sites with loading larger than 0.6 were 4, 7, 16, 20, 41, 50, 51, 53, 54, and 65 (electrodes marked red on the figure of the EGI 65 channel sensor net). Panel **(C)** shows the waveforms of the scalp event-related potentials for the iMMNs for TF3SF3. The blue dotted line indicates the /ɪ/ condition, and the red solid line indicates the /ε/ condition. Time window of statistical analysis is marked with a shaded rectangle.

The interval 260–380 ms was selected (see Figure 3). The permutation ANOVA using condition (/ε/ vs. /ɪ/) and time (six, 20-ms time bins) as the independent variable found that neither main effects nor the interaction was significant (condition: *F*_1_,_192_ = 0.098, *p* = 0.75; time: *F*_5_,_192_ = 1.0626, *p* = 0.38 condition × time: *F*_5_,_192_ = 0.2820, *p* = 0.41).

#### TF1SF5-Derived Measure

**FIGURE 4:**
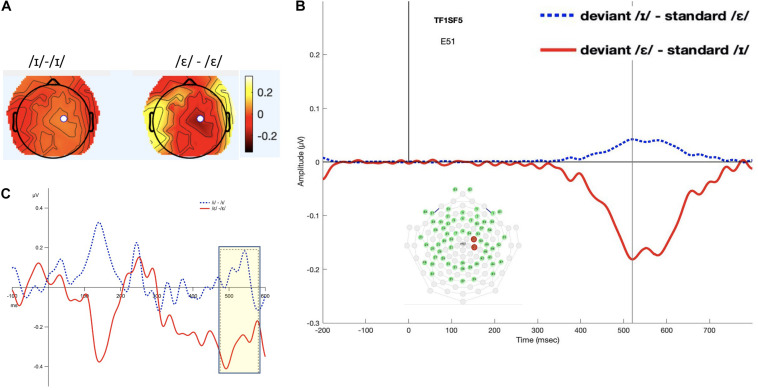
Panel **(A)** shows the topomap of the principal component TF1SF5. Panel **(B)** shows the waveforms of the iMMNs from the principal component TF1SF5. The blue line indicates the /ɪ/ condition, and the red line indicates the /ε/ condition. The PCA component peaked at 520 ms, and the sites with loading larger than 0.6 were 41 and 51 (electrodes marked red on the figure of the EGI 65 channel sensor net). Panel **(C)** shows the waveforms of the scalp event-related potentials for the iMMNs for TF1SF5. The blue dotted line indicates the /ɪ/ condition, and the red solid line indicates the /ε/ condition. Time window of statistical analysis is marked with a shaded rectangle.

The interval 460–580 ms was selected (see Figure 4). The permutation ANOVA using condition (/ε/ vs. /ɪ/) and time (six, 20-ms time bins) as the independent variable revealed a significant main effect of condition (*F*_1_,_192_ = 20.98, *p* < 0.001). The time main effect (*F*_5_,_192_ = 0.2630, *p* = 0.93) and time by condition interaction (*F*_5_,_192_ = 0.1225, *p* = 0.99) were not significant. Follow-up permutation Student’s *t*-test on the condition effect revealed that the amplitude of LDN for /ε/ was significantly more negative than for /ɪ/ (*t* = 3.9447, *p* < 0.001).

### Within-Condition MMN (Deviant /ε/–Standard /ɪ/; Deviant /ɪ/–Standard /ε/)

The analysis approach was identical to the iMMN. Twenty-one temporal factors were retained, which accounted for a total variance of 83.3%. The temporal factors were then submitted for spatial decomposition by using the spatial infomax rotation method. The first three temporal components (TF1, TF2, and TF3) peaked at 650, 298, and 159 ms, and accounted for 36.5, 14.3, and 8.9% of the variance, respectively. No other components had variance accounting for larger than 5%. Only TF3 was in the expected time window of the MMN. Therefore, TF1 and TF2 were discarded from further analysis. Spatial PCA revealed that TF3SF4 showed a spatial pattern over fronto-central regions that was consistent with the MMN topography with the maximal negativity at site 54 (midpoint between Cz and F4) and negativity at inferior posterior regions. The sites with factor loadings >0.6 for TF3SF4 were 4, 41, 50, 51, 53, 54, and 65, and these sites were averaged to derive the measure corresponding to TF3SF4.

#### TF3SF4-Derived Measure

**FIGURE 5:**
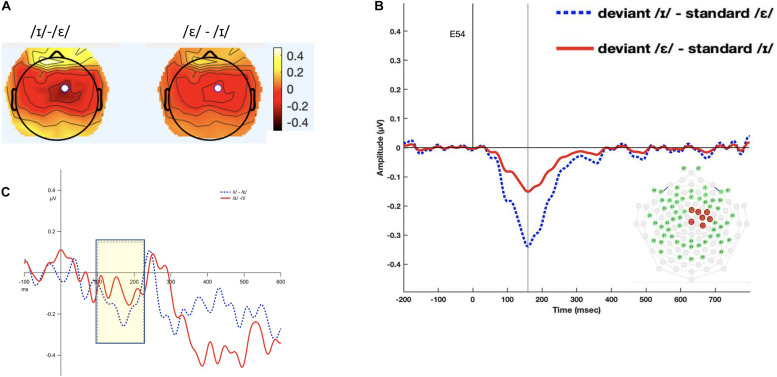
Panel **(A)** shows the topomap of the principal component TF3SF4 for the within-block MMN. Panel **(B)** shows the waveforms of the within-block MMNs from the principal component TF3SF4. The blue line indicates the /ɪ/ condition, and the red line indicates the /ε/ condition. The PCA component peaked at 159 ms, and the sites with loading larger than 0.6 were 4, 41, 50, 51, 53, 54, and 65 (electrodes marked red on the figure of the EGI 65 channel sensor net). Panel **(C)** shows the waveforms of the scalp event-related potentials for the within-block MMNs for TF3SF4. The blue line indicates the /ɪ/ condition, and the red line indicates the /ε/ condition. Time window of statistical analysis is marked with a dashed rectangle.

The interval 100–220 ms was selected (see Figure 5). The results from the permutation ANOVA revealed a significant condition effect (*F*_1_,_192_ = 3.9618, *p* = 0.05). Neither the main effect of time nor the interaction between time and condition was significant (time: *F*_5_,_192_ = 0.3676, *p* = 0.87, condition × time *F*_5_,_192_ = 0.9577, *p* = 0.45). Follow-up permutation Student’s *t*-test on the condition effect revealed that the MMN amplitude was larger when the deviant /ɪ/ was subtracted from /ε/ than when the deviant /ε/ was subtracted from /ɪ/ (*t* = −1.673, *p* = 0.04).

### Order Bias

**FIGURE 6:**
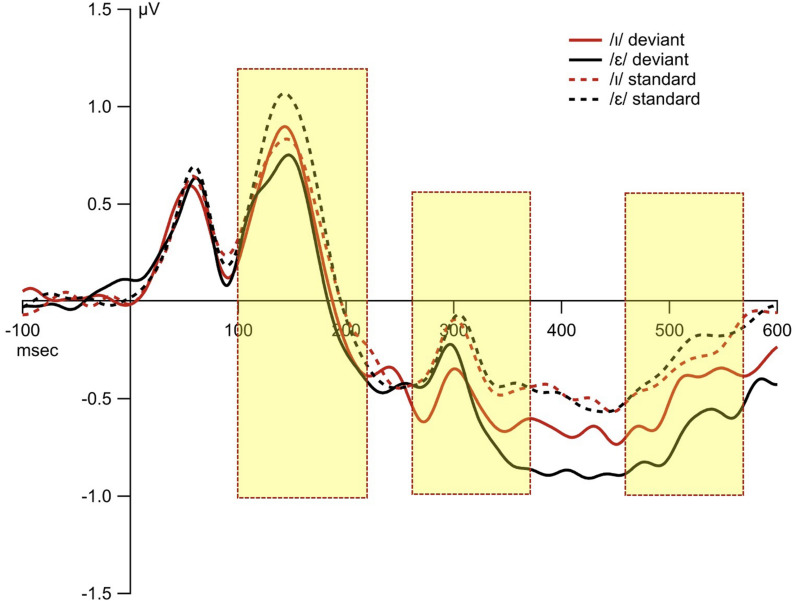
The waveforms from the standard and deviant conditions. The 10 fronto-central sites were chosen based on principal component analysis, and were averaged for display purposes. The three shaded rectangles indicate the time window of statistical analyses based on the principal component analysis.

**FIGURE 7:**
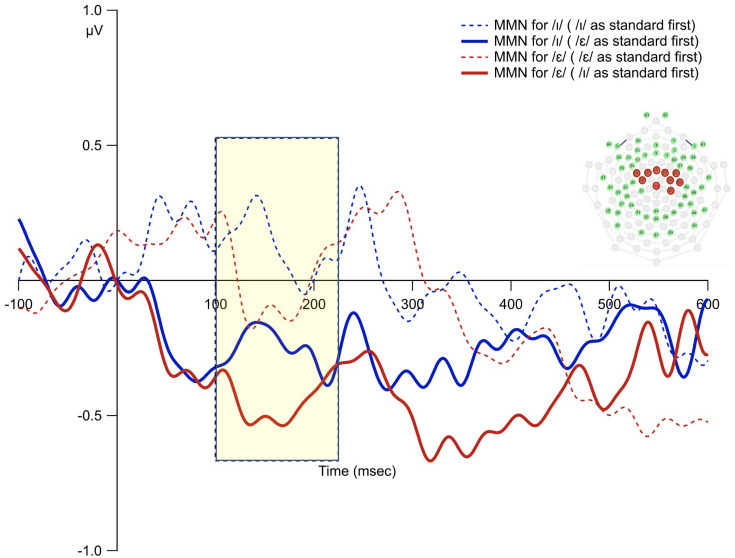
The MMN waveforms from the averages of the 10 fronto-central sites for the subgroup who heard the /ε/ as standard first vs. those who heard the /ɪ/ as standard first. The shaded rectangle indicates time window of analysis based on the principal component analysis.

The overall average waveforms from the two standard and deviant conditions were presented in Figure 6. The first time interval (100–220 ms) was selected to examine order bias effect. The results from permutation ANOVA on the subgroup of participants (*N* = 8) who received /ε/ as the standard condition first showed that deviant /ɪ/ generated larger MMN than deviant /ε/ (*p* < 0.001). However, for the subgroup (*N* = 9) who received /ɪ/ as standard first, deviant /ɪ/ generated smaller MMN than deviant /ε/ (*p* < 0.001). That is, the deviant stimulus that was used as the standard first led to reduced MMN (Figure 7).

## Discussion

### Main Findings

In the present study, we investigated the modulation of the amplitude of MMN to the English vowel contrast /ε/ and /ɪ/ in adult native English speakers using a passive auditory oddball paradigm. We had predicted that the presentation of the less specified vowel /ε/ as the frequent stimulus and the more specified vowel /ε/ as the infrequent stimulus would lead to a smaller MMN than when flipping the stimulus probability for the two vowels (Stemberger, 1991a, b; Scharinger et al., 2012). Our results were consistent with this claim, in the cross-condition comparison, in that both the iMMN and LDN were larger to deviant /ε/ than to deviant /ɪ/ when compared to themselves serving as the standards (deviant /ε/ –standard /ε/; deviant /ɪ/ –standard /ɪ/). However, when comparing the deviant to the within-condition standard (which was a different stimulus), the opposite pattern was observed; specifically, a larger MMN was observed to /ɪ/ as the deviant (minus /ε/ as the standard) than for the reverse of /ε/ as the deviant (minus /ɪ/ as the standard). This within-condition finding is consistent with the NRV model, in which perceptual discrimination is easier when the reference (standard) is a more central vowel. This finding of consistency with both models is not really contradictory because in both calculations the MMN was subtracted from the same standard /ε/. In addition, as predicted, we found an order effect, showing a smaller MMN to the deviant stimulus, if it had previously served as the standard. This order effect, however, did not influence our main findings, because we had roughly equal participants in each order (8 and 9 per order).

### Support for Underspecification

Models of underspecification predict asymmetry of processing and perception. However, one drawback with these models is that the decision with regards to which features are underspecified is often circular. More specifically, underspecified features are proposed to be those that are the least “marked” (e.g., more frequent in the languages of the world, less difficult to learn in child language, perceptually more salient, articulatorily easier, etc.) Often, a feature is declared underspecified because of one or more of these patterns. What is often lacking in these accounts is a unified and consistent proposal for how markedness should be determined (e.g., should it be derived from physical constraints of perception and production or from the language-specific system?) These questions have been grappled with by many linguists (e.g., Haspelmath, 2006; Scharinger and Samuels, 2017), and the current experiment does not provide a solution. Rather, the findings of our study add another piece of evidence showing asymmetries of speech processing that are not easily explained on the basis of acoustic properties.

The stimuli used in the current study were closely matched (via resynthesis and editing) so that the only cues available were the F1 and F2 formants. On a physical scale, both /ɪ/ and /ε/ are centralized compared to the closest tense vowels /i/ and /e/. Thus, on the basis of acoustic-phonetic details, one would predict that these vowels should be equally well-represented at the cortical level, and as a result, we should see no directional difference in the MMN amplitudes for the two vowels. We did, however, see an asymmetry.

Thus, our findings are consistent with a model of underspecification, such as the FUL model (Lahiri and Marslen-Wilson, 1991; Lahiri and Reetz, 2002, 2010). A few fairly recent studies using the MMN measure have also supported the model of underspecification. Hestvik and Durvasula (2016) replicated Phillips et al.’s (2000) asymmetry findings on the consonant voicing contrast /da/ and /ta/. They observed a clear asymmetry with a significant MMN for the /da/ deviant, but not for /ta/ deviant when multiple exemplars of stimuli for each category were used. Similarly, for the LDN, they observed a larger effect for the /da/ deviant compared to the /ta/ deviant. They had predicted this pattern from the claim that English voiceless stops are phonologically specified for spread glottis ([+spread]), and voiced stops are underspecified. Cummings et al. (2017) also found asymmetry of MMN responses for [coronal] underspecification in the English /da/-/ba/ contrast (also Shafer et al., 2004). But this pattern is consistent with an acoustic explanation (Maiste et al., 1995), as well as the underspecification account. However, lending increased support to a phonological account was the finding that Japanese listeners revealed an MMN amplitude asymmetry in the opposite direction of English speakers, using the same stimuli as Hestvik and Durvasula (2016) (Hestvik et al., 2020). This latter finding cannot easily be explained in terms of the acoustic properties of the stimuli. Hestvik et al. (2020) argue that their findings support an underspecification account, but it will also be important to consider the finding in relation to a prototypicality effect.

Our finding that the amplitude of MMN is larger for /ε/ than /ɪ/ was consistent with Scharinger et al. (2012). They observed that MMN was larger to /æ/ as the standard and /ε/ as the deviant and had proposed that /ε/ was underspecified for vowel height, whereas /æ/ and /ɪ/ were specified. Consistency in the pattern for these two studies, however, does not preclude acoustic-phonetic factors underlying this pattern. The finding related to speech production from Stemberger’s (1991b) study provides somewhat stronger support for a linguistic-internal explanation because there is no reason that the asymmetrical pattern should necessarily be consistent across the two different modalities, unless they are linked in some way via phonological representations.

### Support for the NRV Model

Interestingly, our results also can be taken as support for the NRV model (Polka and Bohn, 2003, 2011). In behavioral perception studies, the data are accuracy or response times to a target stimulus. In an oddball paradigm, the target stimulus is the infrequent stimulus. The studies that have observed an asymmetry in perception generally presented stimuli in a habituation paradigm (for infants) or match-to-sample task (stimulus pairs where the participant determines whether the second stimulus is the same or different from the first). Thus, the asymmetry is observed as having higher accuracy scores (or detection of change in the infant studies) when the referent was the more central vowel. The within-condition subtraction (e.g., /ɪ/ minus /ε/) method more directly matches this behavioral paradigm. This method is sometimes used in MMN designs when there is a time limitation (for example infant studies). For the within-condition subtraction in the current study, the MMN amplitude was larger when the deviant was /ɪ/ (/ɪ/ minus /ε/) than when the deviant was /ε/ (/ε/ minus /ɪ/). The NRV posited that vowels with more extreme articulatory-acoustic properties acts as NRVs (e.g., high front vowel /ɪ/ in this experiment), and a vowel change from a more central to a more peripheral position is easier to discriminate than the reversed direction of change. This claim is not necessarily inconsistent with an underspecification account. Specifically, the NRV is consistent with the claim that the peripheral vowels are less marked and could, thus, predict which features are underspecified. Note, however, that the NRV framework has primarily been supported by infant data. Polka and Bohn (2011) emphasized that asymmetrical perceptual bias changes as infants’ language experience increases. Attunement to the first language leads to an attenuation of the default bias favoring peripheral vowels. But given that the perceptual bias favoring vowels in the peripheral spaces is grounded in the acoustic patterns that have an “easy, privileged fit with human auditory/articulatory abilities,” this bias may re-emerge under degraded listening conditions to a native phonemic contrast (Polka and Bohn, 2011).

### Category Goodness

Another explanation that could account for asymmetrical patterns is related to the within-category speech sound structure. Phonemic categories include phonetic variation. Kuhl et al. (1992) proposed that the more prototypical vowel of a speech sound category will be less discriminable from other category members that are less prototypical (Kuhl et al., 1992; Iverson and Kuhl, 1995). In their studies, pairs of stimuli closer to the prototype were more difficult to discriminate than pairs further from the prototype. Other behavioral studies of speech perception have also observed asymmetries in vowel perception. For example, Cowan and Morse (1986) observed better discrimination (in an AX task) of the vowel pair /ɪ/ vs. /i/ when the second in the pair was the more peripheral /i/ in a short interstimulus interval. The effect was greater for longer interstimulus intervals (2-s) between vowels in a pair. They argued that the memory for a vowel was represented as a small, bounded area within the vowel space, and when memory decayed to the first stimulus (A), the representation of the boundary for the vowel space would expand over time, which leads to a shift toward a more centralized vowel. Note that these findings are consistent with the predictions of the NRV model, but that the explanation for the asymmetry is different.

The Kuhl et al. (1992) study also observed a language experience effect in that 6-month-old infants showed a stronger magnet effect for the language-specific prototype of the ambient language (/i/ for Engish and /y/ for Swedish). Thus, language experience influences the internal structure of phoneme categories. It is well-established that early language-specific experience has shaped adult speech perception and neural processing (Näätänen et al., 2007; Shafer et al., accepted). For an underspecification model to show viability, it needs to explain cross-linguistic differences as well as universal patterns (e.g., Hestvik et al., 2020). It is not clear that it can do this better than a prototype model. For example, the pattern of MMN asymmetry observed in Shafer et al. (accepted) showed modulation by language-specific experience (English vs. Spanish first language) where the duration decrement from longer /ɑ:/ as the standard to the shorter /ʌ/ as the deviant showed a larger MMN than the reverse for Spanish listeners, with no amplitude difference observed for American English participants. These results cannot be easily explained in terms of underspecification. Berent et al. (2007) suggested that universal patterns of markedness are visible in non-native listener’s perception. More marked forms are less common across languages. Following this logic, /ʌ/ is more likely to be “marked” than /ɑ:/ because it is the less common vowel across languages and, thus, /ʌ/, as the “specified” form would result in a larger MMN for non-native listeners (all else being equal). The finding with the Spanish listeners, however, did not support this prediction. Rather, the Spanish result is more consistent with a prototype model in which the long interval between repetitions of the American English /ɑ:/ or /ʌ/ (about 1300 ms) resulted in decay in short term memory and the “filling in” of the standard representation with a prototypical Spanish /a/. In consequence, the phonetically more-similar English /ɑ/, as the deviant was not discriminable. In contrast, the American English /ʌ/ as the deviant was sufficiently different from this Spanish vowel representation to allow for discrimination and elicitation of the MMN.

Our finding of an asymmetry in processing could possibly be related to one of the two vowels being a better match to the category prototype. We did not evaluate this in the current study, although identification data from Hisagi et al. (2015a) suggest that the /ɪ/ stimulus used in this MMN study was a better exemplar than the /ε/ stimulus. In this case, the prototype model would predict better discrimination when /ε/ was the standard and /ɪ/ was the deviant. The trace-decay model of Cowan and Morse (1986), in which the English vowel representations shift centrally would also predict better discrimination for this direction.

### Acoustic Explanation

It was less clear that our findings can be taken as consistent with an acoustic-phonetic explanation. However, we cannot completely dismiss the possibility that the higher F2 of /ɪ/ somehow is acoustically more important than the F1. To test this, we would need to use non-speech counterparts with complex tones to examine how the pattern for the higher harmonics modulates MMN. It is clear that psychophysical properties of auditory information do modulate processing (Kirmse et al., 2007; Hisagi et al., 2010), so it is important to fully consider these alternative explanations. Furthermore, some phonetic contrasts are physically more different than others, and given that MMN is larger and earlier to greater physical differences, it is possible that asymmetry is minimized. Thus, the absence of an asymmetry in MMN to /ɪ/ vs. /æ/ in the study by Scharinger et al. (2012) might simply be due to the greater physical difference between this vowel pair. It may also be that psychophysical properties and linguistic experience differentially affect the amplitude vs. latency of the MMN (Näätänen et al., 1997; Shafer et al., accepted). For example, Shafer et al. (accepted) observed an earlier latency of the MMN for an American English vowel duration increment from standard /ʌ/ to deviant /ɑ:/ than for a duration decrement from standard /ɑ:/ to deviant /ʌ/ for English, Japanese and Russian listeners. Only a Spanish group of listeners did not show this latency difference because they had no MMN to the duration increment.

### Other Factors

Group differences in the MMN can be driven by the differences in the standard or deviant alone or both combined. Cummings et al. (2017) found no amplitude differences between the deviant /ba/ and /da/ in the MMN time window, but they did observe a more positive response to the standard /da/ than the standard /ba/. The larger responses were interpreted to indicate a larger neuronal population firing to the coronal place of articulation. Studies of repetition suppression show that repeating a stimulus results in increased positivity of the ERP, which is claimed to be related to a memory trace encoding process (Garrido et al., 2009a, b). It is not clear in what way this notion should be related to underspecification. In the current study, we did not find significant differences between the two standards or between the two deviant stimuli, even though we did see an asymmetry.

The finding of an order effect highlights the importance of examining a range of factors that might influence the study outcome. Specifically, we observed that the amplitude of the MMN was smaller to deviant stimulus if it appeared as the standard in the first block, similar to the finding of Hestvik and Durvasula (2016) and Hestvik et al. (2020). This pattern suggests a lingering memory trace of the deviant when previously presented as the standard. Todd et al. (2014) proposed that the MMN reflects “precision weighted prediction” coding. The MMN responses were primarily decided by the post-synaptic sensitivity of superficial pyramidal cells that encode prediction error. In the flip-flop MMN paradigm, when the standard in the first block was reversed to be the deviant in the second block, the high probability in the first block suppressed the ERP to the deviant in the second block. The reduction of spiking rate to the standard stimuli has been called stimulus-specific adaption. This adaption effect is found at the single-neuron level in both the primary auditory cortex (Ulanovsky et al., 2003, 2004) and in the subcortical structures in animal studies (e.g., Anderson et al., 2009). In a human study, Costa-Faidella et al. (2011) showed that the ERP to a standard after 36 repetitions is more suppressed than that after 24 repetitions. At the same time, there is a concurrent increment in the negativity of the ERP to the deviant (e.g., more negative ERP to the deviant after 36 repetitions than after 24 repetitions). As a result, the MMN can be significantly affected by both short- and long-term stimulus history. What is relevant to the current study is that, if the repetition effect and switch effect are not sensitive to stimulus features, then we would not expect a directional asymmetry.

In addition, McGee et al. (2001) found that MMN amplitudes decline after 10–15 min due to habituation. Our experiment lasted 14 min in each block. It is possible that the habituation effect impacted the MMN amplitudes more generally across the experiment. Irrespective of whether our finding was a primacy or habituation effect, consideration of these order effects is important in this type of design. Future studies are needed better understand whether the long-term repetition factors observed for non-speech stimuli show the same effect for speech. Considering that speech is highly overlearned (Strange, 2011), there is no reason to assume that it will show the same adaptation timecourse as non-speech.

### Limitations

Our study was not designed to directly discriminate among the several competing theories/frameworks discussed above. Our findings provided support for the underspecification, NRV and prototype models. Our study, however, did not test whether listeners exhibited perceptual (behavioral) asymmetries, in discrimination, identification or prototype goodness judgments. Our previous study that tested behavior suggested that /ɪ/ might be closer to the English prototypes than /ε/) (Hisagi et al., 2015a), but to verify this claim it would be necessary to obtain category goodness judgments. It also would have been useful to test a language group for whom these speech sounds would be perceived differently. For example, Hisagi et al. (2015a) found that Spanish listeners were more consistent in labeling /ε/ than /ɪ/ in the identification task, and thus, a reverse asymmetry to that of the English participants would be predicted.

## Conclusion

This study provided additional evidence that asymmetric patterns in speech processing, related to which stimulus can be considered the standard (or referent), are robust. Specifically, the asymmetry observed for the /ɪ/ vs. /ε/ contrast is consistent with other studies. Our findings were consistent with a model of underspecification; however, they were also consistent with other explanations, such as the NRV model or a prototype model. There was little evidence indicating that a purely psychophysical explanation could support the findings. In addition, the finding of an order effect revealed that non-linguistic factors can contribute to asymmetries. Future research needs to examine whether these asymmetries are present early in life and to track these patterns developmentally and in relation to language experience. In addition, to fully test these various models, future studies need to examine a wider range of languages, which have different inventories, such as those with less dense acoustic space vowel inventories (e.g., Mandarin and Japanese).

## Data Availability Statement

The raw data supporting the conclusions of this article will be made available by the authors, without undue reservation.

## Ethics Statement

The studies involving human participants were reviewed and approved by the Institutional Review Board (IRB) of St. John’s University. The patients/participants provided their written informed consent to participate in this study.

## Author Contributions

VS designed the study and created the stimuli. YY helped design and implement the experiment, and collected and analyzed the data supported by research assistants. Both authors wrote the manuscript.

## Conflict of Interest

The authors declare that the research was conducted in the absence of any commercial or financial relationships that could be construed as a potential conflict of interest.
